# Supplementary material for: The interactome of intact mitochondria by cross-linking mass spectrometry provides evidence for coexisting respiratory supercomplexes
Source: Mol Cell Proteomics. 2017 Dec 8;17(2):216–32. doi: 10.1074/mcp.RA117.000470 (PMC5795388; doi:10.1074/mcp.RA117.000470)
Supplement: Supplemental Data [file supp_RA117.000470_133922_0_supp_23978_fzffwf.zip › spectra_annotation/mito_DR_spectra_annotation/355-1-5-1-1-1.pdf]

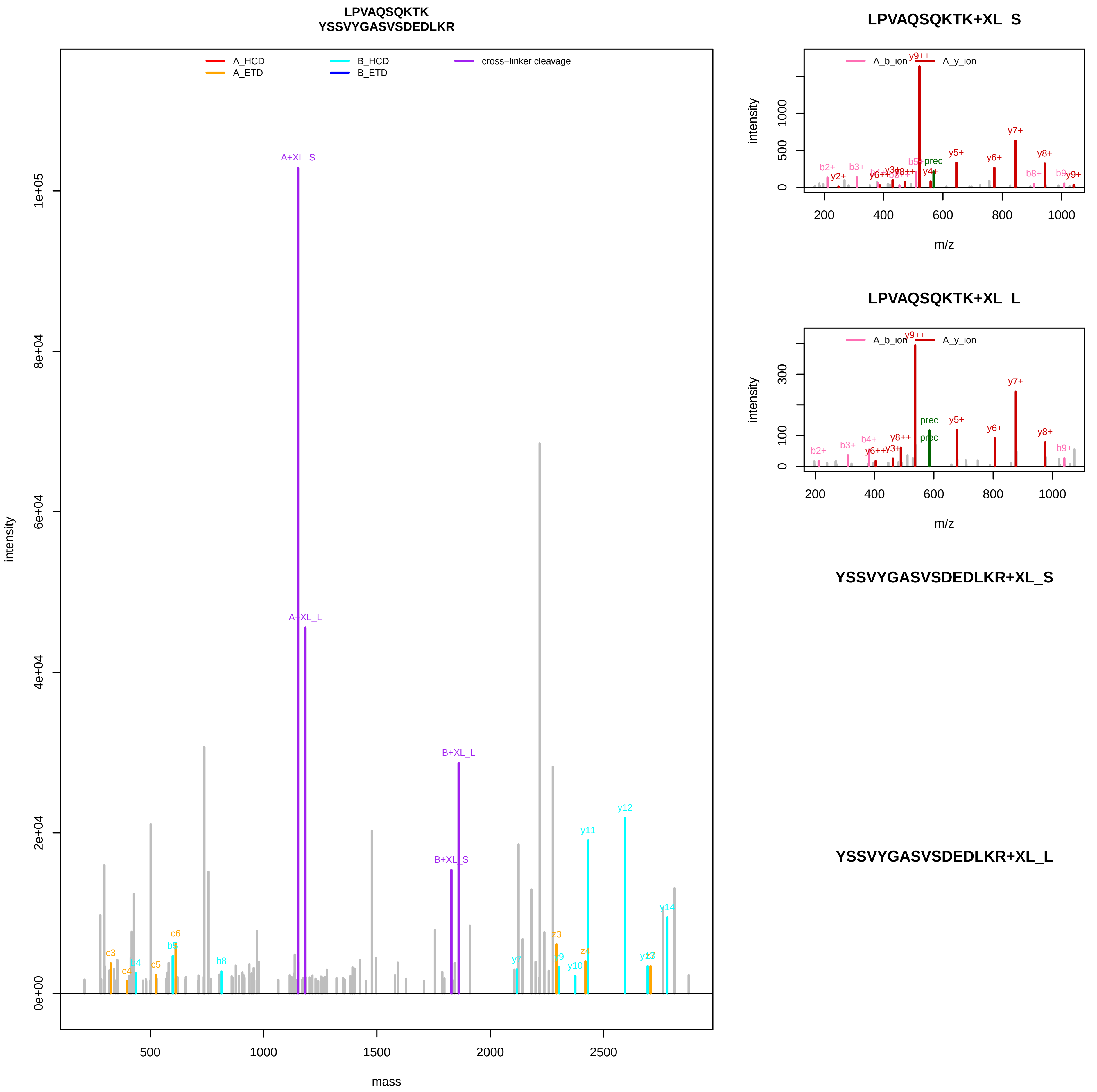
LPVAQSQKTK
YSSVYGASVSDEDLKR
A_HCD
A_ETD
B_HCD
B_ETD
cross−linker cleavage
A+XL_S
A+XL_L
B+XL_L
B+XL_S
c3
c4
b4
c5
b5
c6
b8
y7
y9
z3
y10
z4
y11
y12
y13
z13
y14
intensity
mass
0e+00
2e+04
4e+04
6e+04
8e+04
1e+05
500
1000
1500
2000
2500
LPVAQSQKTK+XL_S
A_b_ion
A_y_ion
y9++
y7+
y5+
y6+
y8+
prec
b2+
y2+
b3+
b5+
y4+
b8+
b9+
y9+
m/z
LPVAQSQKTK+XL_L
y6++
y3+
y8++
b4+
YSSVYGASVSDEDLKR+XL_S
YSSVYGASVSDEDLKR+XL_L
